# Sphingosine-1-Phosphate (S1P) Receptor Modulators for the Treatment of Inflammatory Bowel Disease (IBD): Mechanisms, Clinical Evidence, and Practical Insights

**DOI:** 10.3390/biomedicines13112655

**Published:** 2025-10-29

**Authors:** Natalie Shields, Michael Colwill, Valentina Raspa, Yaw Twum-Danso, Andrew Poullis, Kamal Patel, Sailish Honap

**Affiliations:** 1 Department of Gastroenterology, St George’s University Hospitals, NHS Foundation Trust, London SW17 0QT, UK; natalie.shields@stgeorges.nhs.uk (N.S.); michael.colwill@nhs.net (M.C.); valentina.raspa@stgeorges.nhs.uk (V.R.); andrew.poullis@stgeorges.nhs.uk (A.P.); kamal.patel@stgeorges.nhs.uk (K.P.); 2 Institute of Infection and Immunity, City St George’s, University of London, London SW17 0RE, UK; 3 Department of Gastroenterology, Aneurin Bevan University Health Board, Newport NP18 3XQ, UK; yaw.y.twum-danso@wales.nhs.uk; 4 Department of Immunobiology, School of Immunology and Microbial Sciences, King’s College London, London SE1 9RT, UK

**Keywords:** Sphingosine-1-Phosphate (S1P) Receptor Modulators, ulcerative colitis, Crohn’s disease, inflammatory bowel disease, clinical trials, real world evidence

## Abstract

Inflammatory bowel disease commonly requires advanced therapies to induce and maintain durable remission. Sphingosine-1-phosphate receptor modulators are the latest class of orally administered small molecules that have been added to the therapeutic armamentarium for inflammatory bowel disease. These molecules reduce inflammation by sequestering lymphocytes in lymph nodes, thereby reducing immune cell trafficking to the gut. Etrasimod and ozanimod are both licensed for moderate-to-severe ulcerative colitis and have both shown superiority over placebo, with emerging data for their use in Crohn’s disease. By modulating immune cell distribution, without reducing overall immune function, they offer a highly favourable safety profile. This narrative review explores the pharmacology, safety and efficacy of sphingosine-1-phosphate receptor modulators based on clinical trials and real-world evidence and offers practical guidance on their initiation and monitoring.

## 1. Introduction

Inflammatory bowel disease (IBD) is a chronic immune-mediated inflammatory disease (IMID) that affects the gastrointestinal tract and which untreated can lead to progressive bowel damage requiring surgical intervention [1]. Patients affected by IBD experience recurrent periods of disease relapse, which can significantly impact psychosocial health and quality of life.

IBD primarily consists of ulcerative colitis (UC) and Crohn’s disease (CD), and their treatment has advanced considerably since they were first described in the late 19th century [2]. The broad aims of pharmacological treatment are to induce and maintain remission, reduce disease progression and the need for surgical intervention, and restore quality of life. Standard conventional treatment has historically consisted of 5-aminosalicylic acids, corticosteroids, and immunomodulators such as thiopurines or methotrexate. However, these treatments are ineffective or not tolerated by a significant proportion of patients who subsequently require escalation to an advanced therapy. These therapies include monoclonal antibodies targeting tumour necrosis factor (TNF)-α, interleukin (IL)-12/23, gut-selective integrin antagonists, or small molecule therapies such as Janus-kinase (JAK) inhibitors. Whilst advanced therapies have revolutionized the management of IBD, they have limitations with regard to immunogenicity, side effects, and cost [3]. Furthermore, with the exception of JAK inhibitors, these therapies are administered intravenously or subcutaneously despite studies demonstrating both a patient preference for oral administration and improved treatment adherence [4]. Therefore, there is an evident need for novel safe and effective oral therapeutics to treat IBD.

Sphingosine 1-phosphate (S1P) receptor modulators (S1PRMs) are a class of small-molecule drugs that are an established therapy for multiple sclerosis (MS) [1] and first received regulatory approval for the treatment of UC in 2021 by the Food and Drug Administration (FDA) [2]. Currently ozanimod (Bristol Myers Squibb, Princeton, NJ, United States of America (USA)) and etrasimod (Pfizer, New York City, NY, USA) are licenced in UC, approved by the FDA in 2020 and 2023, respectively, with ongoing clinical trials also investigating the efficacy of tamuzimod (VTX002, Ventyx Biosciences, San Diego, CA, USA) [3] and amiselimod (MT-1303, Mitsubishi Tanabe Pharma, Osaka, Japan) [4]. S1PRMs’ molecular weights of <1 kDa facilitate membrane permeability, reduce manufacturing costs, and may confer additional pharmacokinetic advantages over biologics, including oral bioavailability and absence of immunogenicity [5]. S1PRMs act via a distinct mechanism, selectively modulating lymphocyte egress from lymphoid tissues rather than broadly inhibiting intracellular cytokine signalling [5]. Clinical data have demonstrated encouraging results in terms of rapid response, clinical remission, endoscopic improvement, and sustained efficacy in UC with emerging data in CD [6]. They also appear to have a favourable safety profile, although awareness of potential cardiovascular and ophthalmological side-effects is crucial for safe use.

This review will focus on the mechanistic rationale of using S1PRMs for IBD, the existing evidence to support their use in the treatment of both UC and CD, practical considerations for clinicians including the placement of S1PRMs in the paradigm of IBD treatment, and factors to consider when prescribing these therapies.

## 2. Materials and Methods

A comprehensive literature search was conducted in July 2025. The PubMed database was searched using the terms sphingosine-1-phosphate, sphingosine-1-phosphate receptor modulators, etrasimod, ozanimod, and inflammatory bowel disease. Articles published in English since 2010 were included. Additional references were identified by searching the reference lists of retrieved articles.

## 3. Pharmacology and Mechanism of Action

### 3.1. The S1P Axis

S1P is a membrane-derived lysophospholipid signalling molecule that plays a large role in immune regulation via the S1P receptor (S1PR) [5]. S1P is derived from sphingosine through the action of sphingosine kinases, SphK1 and SphK2, which phosphorylate sphingosine to produce bioactive S1P [5,6].

S1P acts primarily as an extracellular signalling molecule and exerts its function via five different G-protein coupled receptors (S1PR1-5) [5]. S1PRs are expressed in most cells with a high affinity to induce cellular responses in the intestinal mucosa, brain, heart, lungs, and kidneys [7]. These molecules are crucial in the regulation of the immune system as they modulate the circulation of lymphocytes based on the concentration gradient between tissues and the circulatory system [6].

Lymphocyte trafficking is a crucial component of the host immune response [8]. S1PR and S1P interaction mediates the traffic of dendritic, B, and T cells—specifically naïve and central memory T cell that express chemokine receptor 7 (CCR7)—into the systemic circulation and other tissues [8]. The traffic is mediated by the concentration gradient of S1P between lymphoid organs and surrounding tissues [6]. Movement of lymphocytes out of lymphoid organs results in inflammation via downstream mediator cytokines, including interferons, interleukins, and TNFs [6]. S1PRMs bind to the S1PRs, initiating a signalling cascade that leads to internalization of the S1PR and causes degradation of intracellular S1P, resulting in decreased lymphocyte egress and ultimately decreased downstream inflammation [9]. Lymphocyte migration from the systemic circulation to the gut mucosa is known to be a key determinant of inflammation in the complex pathophysiology of IBD [10,11], hence the theoretical rationale of using S1PRM to treat IBD.

#### S1P Receptor Subtypes

Various S1PR expression patterns are present within the immune system, and this, along with their individual functions, is summarised in Table 1. S1PR1-3s are the most widely expressed throughout the body, with S1PR1 specifically responsible for lymphocyte egress from lymphoid tissues into the systemic circulation, while S1PR4-5s are expressed in a more limited fashion, and this variable expression offers an opportunity for selective targeting [9].

Target specificity is a critical component in developing a safe, effective, and well-tolerated medication. By targeting specific S1PRs, off-target effects can be avoided, and therapeutic effects can be maximized. The modulation of S1PR1 has been shown to regulate the lymphocytic migration without downregulating overall immune function, in contrast to other immunosuppressants historically used for IBD [12], and therefore has a favourable side-effect profile [13].

### 3.2. Mechanism of Action of S1PR Modulators

S1PRMs initially bind to S1PR as agonists; however, prolonged exposure leads to S1PR internalization and degradation, rendering lymphocytes unresponsive to the S1P gradient that normally governs their egress from lymph nodes. This results in the selective sequestration of naïve and central memory T cells, which express both S1PR1 and the lymph-node-homing receptor CCR7 [8,14]. The reduction in circulating lymphocytes limits the recruitment of inflammatory cells to target tissues, such as the central nervous system in MS [13] and the colonic mucosa in IBD [8].

**Figure 1:**
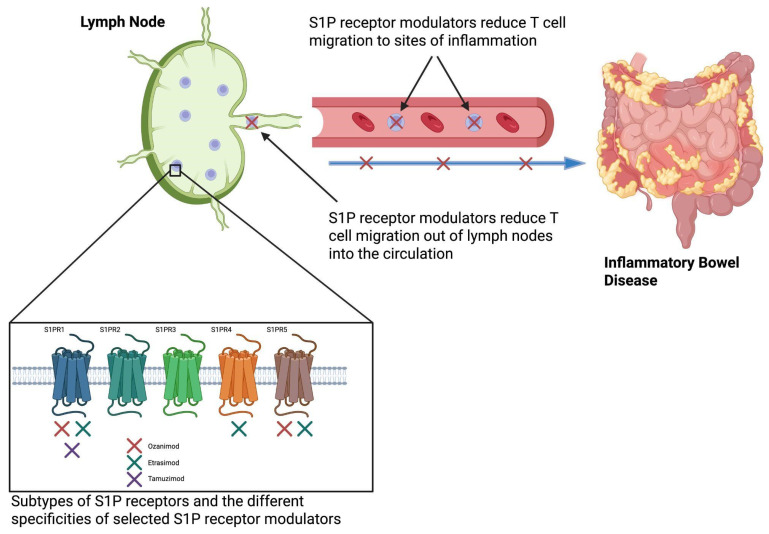
Etrasimod modulates S1PRs to inhibit T cell egress from lymph nodes, thus reducing their migration to sites of inflammation, such as the colonic mucosa in ulcerative colitis [17]. Created in BioRender. S1P: Sphingosine-1-phosphate. Colwill, M. (2025) https://BioRender.com/p02otab (accessed on 1 September 2025).

Etrasimod and ozanimod, two S1PRMs licensed for the treatment of UC, differ in S1PR subtype selectivity, leading to differences in immunologic effects and clinical profiles. Ozanimod, the first S1PRM for UC, which is metabolized into seven active plasma metabolites, including the two major active metabolites CC112273 and CC1084037 [15], selectively targets S1PR1 and S1PR5, offering focused modulation of lymphocyte trafficking with potential neuroprotective effects via S1PR5 in oligodendrocytes [16,17]. In contrast, etrasimod exhibits a broader receptor profile, modulating S1PR1, S1PR4, and S1PR5 [18] (Figure 1). The additional activity at S1PR4, a receptor expressed on dendritic cells and other innate immune cells, suggests that etrasimod may influence cytokine signalling pathways and innate immune responses more broadly than ozanimod.

## 4. Historical Development of S1PR Modulators

### 4.1. Fingolimod

The therapeutic potential of targeting S1PR in inflammatory conditions was first demonstrated in clinical studies of fingolimod (Novartis, Basel, Switzerland), a non-selective S1PRM, for the treatment of relapsing–remitting MS. Fingolimod binds to S1PR1,3,4,5 with high affinity and was shown to reduce lymphocyte circulation by 70% [6]. Its non-selective nature may explain the broad range of adverse events (AEs) reported throughout its initial clinical trials, including bradyarrhythmia and atrioventricular (AV) block [19,20]. Whilst approved by the FDA for the treatment of relapsing–remitting MS in 2010, it was already receiving attention for its potential use in the treatment of IBD in the early 2000s, when studies suggested success in ameliorating chronic colitis in animal models [9]. However, due to concerns over the described side effects, particularly an unfavourable cardiac safety profile, fingolimod has never undergone human trials as a treatment for IBD. It became evident that there was a need for a novel, more selective S1PRMs in order to reduce off-target effects and create safer pharmacological profiles while preserving efficacy.

### 4.2. Ozanimod

Initial phase 1 studies of ozanimod were conducted by Receptos (now acquired by Celgene) in the early 2010s, with results demonstrating that ozanimod was well-tolerated, induced dose-dependent lymphocyte reduction, and had a more favourable safety profile compared to its predecessor fingolimod [20]. The subsequent clinical trials RADIANCE and SUNBEAM were run in parallel and demonstrated ozanimod’s efficacy in the treatment of MS [21]. Shortly after the success of the MS studies, the first proof-of-concept study for ozanimod in UC-TOUCHSTONE [22] was conducted on patients with moderate-to-severe disease followed by the phase 3 TRUE NORTH study [23], which demonstrated superior efficacy over placebo for inducing remission during both induction (18.4% vs. 6.0%, *p* < 0.001) and maintenance (37.0% vs. 18.5% *p* < 0.001). It received FDA approval in 2020 and European Medicines Agency (EMA) approval in 2021 under the trade name Zeposia [24].

### 4.3. Etrasimod

Etrasimod was developed by Arena Pharmaceuticals before being acquired by Pfizer in 2022 [25]. An initial dose-range-finding study, entitled OASIS, proved that a once-daily 2 mg dose was sufficient in meeting the study’s primary and secondary endpoints [26]. Two subsequent phase 3 double-blind randomized control studies, ELEVATE-UC 12 and ELEVATE-UC 52, were run in a treat-through design, which ultimately led to approval by the FDA and EMA in 2023 under the trade name Velsipity for use in moderate-to-severe UC after failure or intolerance of conventional therapies [27]. Etrasimod’s receptor selectivity is thought to contribute to its safety profile, as S1PR1,4,5 are all primarily associated with immune regulation, whilst subtypes S1P2 and S1P3, which it does not modulate, act on cardiovascular and pulmonary tissue, potentially leading to adverse effects, discussed further below [28]. The key pharmacokinetic and pharmacodynamic properties of these therapies are summarized in Table 2.

## 5. Evidence of Efficacy and Safety in UC

### 5.1. Randomized Clinical Trials Evaluating Ozanimod

Ozanimod is a more selective S1PRM than its first-generation predecessor fingolimod, binding to S1PR1 and S1PR5 with high affinity [15], and was the first S1PRM to be approved to treat moderate-to-severe UC in 2021.

TOUCHSTONE was a phase 2, double-blind, placebo-controlled trial of ozanimod in 197 adults with moderate-to-severe UC (Mayo score 6–12, endoscopic sub-score 2–3) [22]. Patients were randomized 1:1:1 to ozanimod 0.5 mg, 1 mg, or placebo for 8 weeks. Responders continued with a 32-week extension; non-responders at week 8 could start open-label ozanimod. At week 8, clinical remission (Mayo ≤ 2, no endoscopic sub-score > 1) occurred in 14% (0.5 mg), 16% (1 mg), and 6% (placebo), with significance for 1 mg vs. placebo (*p* = 0.048). By week 32, remission rates rose to 26%, 21%, and 6%, respectively. AEs were generally mild (headache, anaemia). One patient (0.5 mg arm) with pre-existing bradycardia experienced transient, asymptomatic sinus bradycardia and atrioventricular (AV) block, resolving without intervention before treatment discontinuation [22].

**Figure 2:**
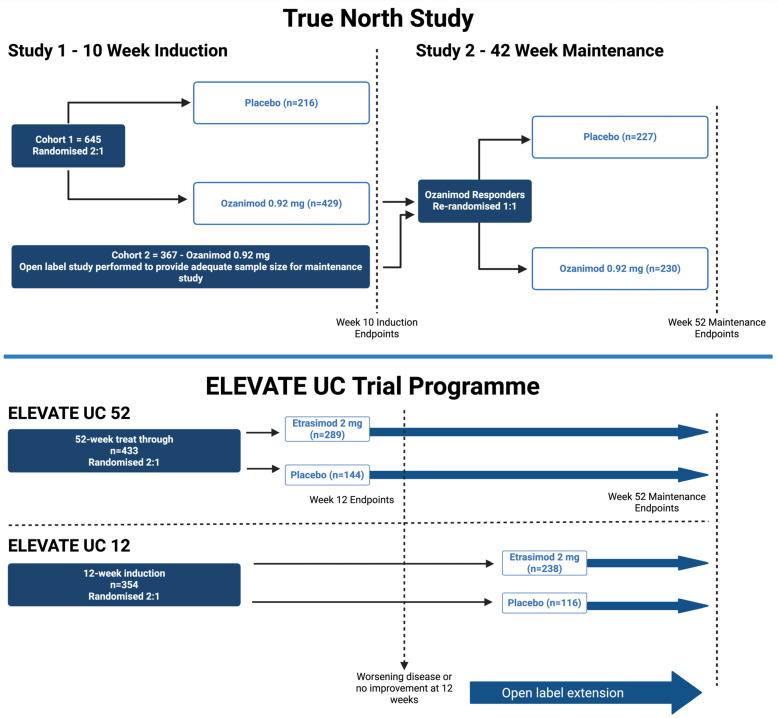
Schematic of the pivotal phase 3 trials in inflammatory bowel disease for ozanimod (TRUE NORTH—re-randomized design) and etrasimod (ELEVATE—treat-through design). Created in BioRender. Colwill, M. (2025) https://BioRender.com/3q476cb (accessed on 1 September 2025).

TRUE NORTH was a phase 3, multicentre, randomized, double-blind, placebo-controlled trial evaluating ozanimod for induction and maintenance in moderate-to-severe UC (Table 3) [23]. Patients in cohort 1 were randomized 2:1 (Figure 2) to ozanimod 0.92 mg (*n* = 429) or placebo (*n* = 216) or received open-label ozanimod (cohort 2, *n* = 367), with a 7-day dose escalation to reduce bradycardia risk. At week 10, responders were re-randomized to ozanimod or placebo for 42 weeks. Clinical remission was higher with ozanimod vs. placebo at the end of the 10-week induction (18.4% vs. 6.0%; *p* < 0.001) and 42-week maintenance (37.0% vs. 18.5%; *p* < 0.001). Clinical response was also superior in the induction (47.8% vs. 25.9%; *p* < 0.001) and maintenance arms (60.0% vs. 41.0%; *p* < 0.001). AEs were more frequent with ozanimod than placebo, though non-serious infection rates were similar. Bradycardia occurred more often with ozanimod in the induction cohort; however, there was no difference from placebo during the maintenance study [23].

### 5.2. Real-World Evidence on Ozanimod

Whilst these trials confirmed ozanimod’s efficacy in UC, real-world evidence (RWE) has added insights on safety, disease burden, and outcomes in more diverse settings. A prospective, observational cohort study from the University of Chicago with 45 patients showed clinical remission in 53% at week 10 and 25% at one year in a treatment-refractory cohort (76% anti-TNF–exposed) [30]. A larger TriNetX retrospective study found similar 12-month corticosteroid-sparing effects to vedolizumab but higher therapy-switch rates with ozanimod [31]. While broadly supporting phase 3 findings, questions remain on long-term efficacy versus other advanced therapies, and further data are required.

### 5.3. Randomized Clinical Trials Evaluating Etrasimod

With regard to etrasimod, the pivotal studies that demonstrated its efficacy in UC were OASIS, ELEVATE-UC 12, and ELEVATE-UC 52 [32,33]. The key findings from these studies are summarized in Table 4.

OASIS was a phase 2, double-blind, randomized trial evaluating etrasimod in 156 adults with moderate-to-severe UC [26]. Patients received oral etrasimod at 1 mg or 2 mg or placebo once daily for 12 weeks. The primary endpoint was the change in modified Mayo score (stool frequency, rectal bleeding, endoscopy) from baseline to week 12. Etrasimoda at 2 mg daily met the primary endpoint with greater clinical improvement compared to placebo (*p* = 0.009), while 1 mg daily showed a non-significant trend (*p* = 0.15). Clinical remission (an exploratory endpoint) at week 12 occurred in 33% (2 mg) vs. 8.1% (placebo, *p* < 0.001). Safety data were reassuring, with a discontinuation rate of less than 10%. AEs occurred in 55.1%, but only 7.7% were considered to be drug-related. Common AEs were worsening UC, upper respiratory infection, nasopharyngitis, and anaemia. Three patients had asymptomatic, transient cardiac conduction changes after the first dose, all of whom had pre-existing AV block on pre-dosing electrocardiogram (ECG) [26].

ELEVATE-UC 12 and 52 were phase 3, multicentre, randomized, double-blind, placebo-controlled trials assessing etrasimod 2 mg daily vs. placebo in adults with moderate-to-severe UC (*n* = 354 and *n* = 433, respectively, Figure 2) [33]. Both trials used a treat-through design to assess efficacy at 12 and 52 weeks. ELEVATE-UC 12 consisted of a 12-week induction with a subsequent optional open-label extension (OLE), whilst ELEVATE-UC 52 involved a 12-week induction, at which point non-responders could enter an OLE, followed by 40-week maintenance programme [33].

In ELEVATE-UC 52, remission at week 12 was higher with etrasimod compared to placebo (27% vs. 7%; *p* = 0.0001), and sustained remission at week 52 remained superior (32% vs. 7%; *p* < 0.0001) [33]. In ELEVATE-UC 12, remission at week 12 was 25% compared to 15% with placebo (*p* = 0.026). Safety was favourable and consistent with prior studies [33].

The ELEVATE studies also included patients with isolated proctitis, a sub-group who are usually excluded from randomized clinical trials, and a post hoc analysis of these patients showed superiority over placebo for clinical remission at weeks 12 (42.9% vs. 13.6%) and 52 (44.4% vs. 11.1%), endoscopic improvement (52.4% vs. 22.7%) at week 12, and bowel urgency numerical rating scale score at week 12 (all *p* < 0.01) [34].

In ELEVATE-UC 52, AEs occurred in 71% (etrasimod) compared to 56% (placebo); in ELEVATE-UC 12, it was 47% in both groups. Across both ELEVATE studies, there were nine cases (1.7%) of bradycardia reported [33]. Two of these cases were symptomatic and resulted in discontinuation from the study, and eight of these cases were reported upon the first day of dosing (the remaining case was reported on day 2). Three cases of AV block were reported; two of the cases had resolved following treatment discontinuation, and the other was identified prior to the first dose. Macular oedema occurred in three patients, two of which were in the etrasimod group of either study (one of which had pre-existing uveitis). All events of macular oedema resolved with discontinuation of the drug [33].

In addition, ENLIGHT-UC, a randomized, double-blind, placebo-controlled, multicentre, phase 3 study performed in 606 patients with moderate-to-severe UC in East Asia demonstrated that etrasimod at 2 mg daily was superior to placebo for both induction of remission at week 12 (*n* = 57 [25·0%] vs. 6 [5·4%]; adjusted difference 20·4%; 95% CI 13·4–27·4%; *p* < 0·0001) and maintenance of remission at week 40 (37 [48·1%] vs. 10 [12·5%]; adjusted difference 35·9%; 95% CI 22·5–49·2%; *p* < 0·0001) [35]. Etrasimod was well tolerated with 5 (2%) of 228 patients treated with etrasimod and 4 (4%) of 112 patients treated with placebo discontinuing study treatment due to treatment-emergent AEs (TEAE) during the induction period, and 1 (1%) of 77 patients treated with etrasimod and 1 (1%) of 81 patients treated with placebo discontinuing study treatment due to TEAEs during the maintenance period. Safety profiles were similar to the ELEVATE trial programme.

It should also be noted that whilst the total sample sizes from the OASIS, ELEVATE-UC 12, and ELEVATE-UC 52 studies on etrasimod did not meet the numbers required by universal vaccine and drug development standards [36], they were within the accepted range for pivotal trials in IBD and were based upon determinations of statistical power needed to detect clinically meaningful differences in efficacy and safety outcomes.

### 5.4. Real-World Evidence on Etrasimod

As of the time of writing, only one real-world study of etrasimod has been published. A tertiary IBD centre in Chicago reported early experience in 22 patients with moderate-to-severe UC (82% 5-aminosalicylic-acid-exposed, 23% advanced-therapy-exposed) [37]. In this observational, cohort study at week 12, 64% achieved remission, but discontinuation was high, with only 12 patients remaining on therapy at week 26; of these, approximately half maintained remission. Two ongoing observational trials—ENDEVOUR-UC and EFFECT-UC—will follow larger UC populations for up to 52 weeks, assessing clinical remission at weeks 12 and 52 via remote symptom tracking and patient-reported outcomes. ENDEVOUR-UC is expected to report in 2025, EFFECT-UC in 2027 [38,39].

## 6. Evidence of Efficacy and Safety in CD

### 6.1. Ozanimod for Crohn’s Disease

Ozanimod has been investigated for the treatment of CD in the STEPSTONE and YELLOWSTONE trials [40,41]. STEPSTONE was a 12-week, open-label, phase 2 trial in 69 patients with moderate-to-severe CD and inadequate response or intolerance to corticosteroids, immunosuppressants, or biologics. After a 7-day dose escalation from 0.25 mg to 1 mg daily, patients maintained the 1 mg dose for the study and 100-week extension period. The primary endpoint was a change in the Simple Endoscopic Score for CD (SES-CD) from baseline to week 12. Mean SES-CD change was −2.2, with similar reductions in bio-naïve and bio-exposed patients. Endoscopic response (≥0% SES-CD reduction) occurred in 23.2% and remission (SES-CD ≤ 4 with additional criteria) in 10.1% [40].

Ozanimod was generally well tolerated; most AEs were mild-to-moderate and related to underlying CD such as abdominal pain or arthralgia. Two patients developed abdominal abscesses, and three experienced serious treatment-emergent AEs from fistula complications, all after treatment discontinuation. No clinically significant first-dose heart rate changes were observed, likely due to gradual titration. Although limited by a small sample size and lack of a control group, STEPSTONE suggested ozanimod may be effective for CD [40].

The open-label extension (OLE) of the phase 3 YELLOWSTONE trial was designed as a randomized, double-blind, placebo-controlled trial with a larger patient cohort (*n* = ~600) [41]. Patients were randomized 2:1 to receive either ozanimod 0.92 once daily (after a 7-day dose escalation) or placebo for the 12-week induction period before progressing to either the OLE or the 52-week maintenance phase [41]. For the induction study, the primary endpoint was clinical remission at week 12 (evaluated by the Crohn’s Disease Activity Index (CDAI)). The study was terminated by Bristol Myers Squibb after failing to reach the primary endpoint [42].

Therefore, despite the findings of the STEPSTONE trial, the current available data suggests that ozanimod is not effective for the treatment of moderate-to-severe CD.

### 6.2. Etrasimod for Crohn’s Disease

The CULTIVATE trial (*n* = 375) is a phase 2/3, multicentre, randomized, double-blind parallel group study, comprising five substudies, that aims to assess etrasimod in moderate-to-severe CD refractory or intolerant to one or more conventional or advanced therapies [43]. Completed in June 2025, only induction results from Substudy A are available at the time of writing. Substudy A was a 14-week, phase 2 dose-ranging study followed by 52-week maintenance, randomizing 83 patients 1:1 to etrasimod 2 mg or 3 mg daily [43]. The primary endpoint—endoscopic response at week 14 (defined as endoscopic remission (SES-CD ≤ 4 and ≥2-point reduction from baseline with no subscore >1) or ≥50% decrease in SES-CD score)—was achieved by 21.4% (2 mg) and 9.8% (3 mg) [43]. Etrasimod was generally well tolerated; AEs were more frequent with 3 mg, most commonly headache, arthralgia, and fatigue. Two patients in the 3 mg group developed asymptomatic Mobitz type 1 AV block during titration, consistent with UC trial findings. Serious adverse events (SAEs) occurred in 4.8% (2 mg) and 2.4% (3 mg) [43].

Whilst only limited data are currently available, substudy A of CULTIVATE suggests that treatment with etrasimod 2 mg and 3 mg may induce an endoscopic response and clinical improvement in patients with moderate-to-severe CD. The small sample size and lack of control group means that it is not possible to draw any firm conclusions currently until the full results of the CULTIVATE study are available.

## 7. Special Populations

### 7.1. The Elderly

The incidence and prevalence of IBD in the elderly, classed as those over 60 years old, is increasing worldwide, and this poses challenges with regard to the management of IBD due to the higher rates of comorbidity, polypharmacy, and mortality [44]. There is also data suggesting that this population is more likely to suffer from treatment-related complications, such as infections and death [45], and there is therefore a need for safe and efficacious treatments suitable for this population [46].

S1PRMs have been shown to have a favourable safety profile in the previously described trials in IBD, with a systematic review finding a lower rate of SAEs for all S1PRMs compared to placebo in a pooled analysis (Relative Risk (RR)—1.25; 95% CI 0.80–1.94, *p* = 0.33) [47]. This study also found no significant difference when comparing etrasimod and ozanimod individually, suggesting that the favourable safety profile, albeit with recognized SAEs, is a common trait across the drug class.

Given the complicating factors seen in the elderly population as described, dedicated data is required to understand the safety and efficacy of S1PRM in this population. The TRUE NORTH study described earlier included patients up to the age of 75, and a post hoc analysis in patients over the age of 60 (*n* = 83) found no difference in SAEs or efficacy compared to younger populations [48]. Two malignancies were identified, but neither was in the over-60 cohort. A similar post hoc analysis of the ELEVATE UC trials, which included adults up to the age of 80, again found similar rates of SAEs in those aged over 60 compared to younger patients [49]. Noteworthy was the lack of any malignancy or deaths in the trial period, although this may be partially explained by the relatively short follow-up period of 52 weeks. Rates of serious infection were similar regardless of age, and there were no significant differences in herpes zoster infection rates. The study also found that etrasimod was associated with significantly greater improvements in rates of clinical remission, clinical response, symptomatic remission, endoscopic improvement, endoscopic-improvement–histologic-remission, histologic–endoscopic mucosal improvement, and corticosteroid-free remission than placebo, regardless of the age of the patient [49].

Polypharmacy is more common in the elderly, and this has important implications for S1PRM [50]. Both etrasimod and ozanimod are extensively metabolized by the cytochrome P450 proteins in the liver, specifically CYP3A4, CYP2C8, and CYP2C9 [51,52]. Therefore, medications that inhibit these enzymes, such as omeprazole, clarithromycin, or amiodarone, may result in toxicity, whilst co-prescription with inducers of these enzymes, such as carbamazepine or St. John’s wort, could result in rapid catabolism of S1PRM, reducing clinical efficacy.

### 7.2. Cardiovascular Disease

A meta-analysis found that S1PRM use was associated with cardiovascular AEs in 10.9% of patients with MS; this included any of arrhythmia, bradyarrhythmia, tachyarrhythmia, hypertension, hypotension, heart failure, coronary artery disease, acute coronary syndrome, or chronic coronary syndrome [53]. Whilst cardiovascular side-effects do appear to be a class effect, it should be noted that this study included data from several different agents, including fingolimod, ponesimod, and siponimod, which are not licensed for the treatment of IBD.

The phase 2 and 3 clinical trials described above also identified cardiovascular side effects when treating patients with IBD. Symptomatic bradycardia occurred in five (0.63%) patients treated with ozanimod in the TRUE NORTH study, but none developed second- or third-degree AV block [23]. Of these five, one was symptomatic, and all saw resolution of the bradycardia with treatment cessation. Across the OASIS, ELEVATE UC and OLE trials, there were 14 patients (1.5%) who developed bradycardia, all of whom were in the etrasimod cohorts [54]. Two were symptomatic, and a total of four required treatment discontinuation. Twelve patients developed bradycardia on day 1, and two developed it on day 2 of treatment. There were seven cases of AV block—six with first-degree AV block and one with Mobitz type 1 s-degree AV block, all of which resolved after discontinuation of etrasimod [54]. Corrected Q-T interval (QTc) prolongation, which was seen in fingolimod, ponesimod and siponimod for MS treatment, was not seen in ozanimod or etrasimod.

Patients in the ELEVATE UC trials treated with etrasimod were also more likely to develop hypertension. Whilst 11 patients (2%) developed hypertension in the etrasimod cohort compared to 2 (0.7%) in the placebo group, all of these incidences were mild and did not lead to treatment discontinuation [33]. In the TRUE NORTH studies, similar rates of new-onset hypertension were seen in patients treated with ozanimod, and one patient in each of the induction and maintenance phase studies developed a hypertensive crisis; both instances occurred on day 1 and resolved without treatment discontinuation. There were no other cardiovascular AEs seen in the trials including myocardial infarctions or heart failure [23].

### 7.3. Liver Disease

In both the ELEVATE and TRUE NORTH trial programmes, elevation in liver enzymes was more common in those treated with an S1PRM compared to placebo [23,33]. Furthermore, 2.7% of patients treated with ozanimod developed elevations in alanine aminotransferase (ALT) compared to 0.2% in the placebo, and whilst no patients met the criteria for Hy’s law, treatment was discontinued in four patients (0.4%). Similarly, in the ELEVATE programme, whilst no patients met Hy’s law, rises in ALT were seen in 10% in the induction cohort and 16% in the maintenance cohort of those treated with etrasimod with 2 patients subsequently discontinuing the trial [23,33].

Whilst in both trial programmes, patients with severe hepatic impairment or deranged liver function tests were excluded, the available evidence suggests that etrasimod and ozanimod are both safe in those with mild or moderate hepatic impairment. No dose adjustment is required for patients with mild to moderate liver disease; however, in Child–Pugh C cirrhotic patients, S1PRMs should be avoided. Follow-up and real-world clinical studies are needed to assess for longer-term outcomes in hepatic impairment.

## 8. Other Agents

Several S1PRMs failed to reach the market due to a combination of clinical and strategic factors, offering important lessons for future drug development. Amiselimod was a second-generation S1PRM engineered to selectively target S1PR1 and S1PR5 and has demonstrated a favourable cardiac safety profile due to this selectivity [55]. Clinical trials were initially halted by Biogen due to strategic market concerns; however, with increased interest in S1PRMs in treating IBD and IMIDs, these have now been restarted by Mitubishi Tanabe, and recruitment in phase 2 trials in UC has been completed, with initial data showing superiority over placebo for mucosal and histological improvements in mild–moderate disease [56]. Ceralifimod (ONO-4641, Ono Pharmaceutical Company, Osaka, Japan and Merck KGaA, Darmsladt, Germany), showed promise in early trials for multiple sclerosis, but trials investigating its use as an IBD therapy were also discontinued for strategic business reasons [57].

Tamuzimod is a selective S1PRM that acts on S1PR1 and has completed phase two trials in UC. These trials demonstrated that tamuzimod at both 30 mg (*p* = 0.041) and 60 mg (*p* = 0.018) was associated with significantly higher rates of remission at 13 weeks compared to placebo [3]. There were no cardiovascular-related AEs reported and no cases of macular oedema. Phase 3 trials are awaited.

## 9. Practical Considerations for Clinicians

### 9.1. Treatment Positioning

There is no direct evidence from clinical trials to guide positioning of S1PRMs in the treatment pathway. Multiple factors should be considered by treating physicians, including prior advanced therapy exposure, comorbidities, and extra-intestinal manifestations.

A key factor that makes S1PRMs an attractive option for treating patients with IBD is the oral route of administration, which may be preferable for patients and reduces healthcare service utilization and cost from infusion suite bookings. Pharmacokinetic benefits of S1PRMs also include a lack of immunogenicity, a lack of weight-based dosing, and a rapid onset of action.

There are no direct comparator trials with regard to efficacy, and therefore positioning different agents in a treatment algorithm is challenging and relies largely upon network meta-analysis (NMA). An NMA from 2024 found that in advanced therapy, for naïve patients with UC, etrasimod at 2 mg was comparable in both induction and maintenance phases to other agents, with the exception of upadacitinib [58]. A separate NMA by Jairath et al. found that whilst effective in both advanced-therapy-naïve and -exposed cohorts, etrasimod and ozanimod showed considerably lower rates of clinical response and clinical remission in the advanced-therapy-exposed cohort [59]. A post hoc analysis of the ELEVATE studies also showed lower rates of clinical remission and endoscopic response in thiopurine-exposed patients [60]. The American Gastroenterological Association has also classed both drugs as having ‘lower efficacy’ in advanced-therapy-exposed patients [61]. Similar data for CD are not currently available.

With regard to safety, comparisons to other advanced therapies are also difficult due to a lack of available head-to-head data. As described, the safety profile of S1PRMs is generally favourable, particularly with regard to their low infection risk, including herpes zoster, rates of malignancy, and cardiac AEs [27]. In comparison, anti-TNF agents have been associated with an increased risk of infection, lymphomas, and melanomas [62]; JAK inhibitors have been associated with cardiac AEs, venous thromboembolism, herpes zoster and a possible increased risk of malignancy [63]; and ustekinumab, vedolizumab, and the p19 inhibitors appear to pose a smaller infective or malignant risk [64]. S1PRMs may therefore be an appropriate choice where other agents are contraindicated due to concerns about potential AEs with decisions made on an individual basis.

Given their favourable safety profile and drug characteristics as described, as well as the available efficacy data, it is the opinion of the authors that S1PRMs are best positioned for use after failure or loss of response to conventional therapies in UC (e.g., 5-aminosalicylic acid, steroids or immunomodulators). There is insufficient data to make recommendations in CD.

### 9.2. Initiating S1PRMs

Prior to treatment initiation, all patients should be assessed with

- Full blood count (FBC), renal and liver profile (within the preceding 6 months).
- Pre-immunosuppression infection screening—testing for serological evidence of current or previous infection with Hepatitis B and C; Human Immunodeficiency Virus; previous varicella exposure; tuberculosis.
- A full vaccination history to ensure these are up to date.
- A plan for contraception in females of childbearing age.
- Baseline ECG to assess for evidence of conduction abnormalities, pre-existing bradycardia or ischaemic changes (see below).
- Ophthalmological assessment for macular oedema (see below).
- A full drug history—S1PRMs should not be co-administered with monoamine oxidase inhibitors or moderate/strong inhibitors of CYP2C8, CYP2C9, and CYP3A4.

### 9.3. Dosing and Titration

Ozanimod requires dose titration in the first week, with 0.23 mg administered once a day (OD) from days 1 to 4, then 0.46 mg (OD) from days 5 to 7, and then 0.92 mg OD from day 8 onwards. Etrasimod is dosed at 2 mg once a day and requires no dose titration.

### 9.4. Cardiac Monitoring

S1PR1 are found on cardiac myocytes and when activated lead to an intracellular shift of potassium disrupting cardiac rhythm. A safety analysis by Vermeire et al. found an increased risk of bradycardia in those taking beta-blockers (Hazard Ratio 4.77 (95% CI: 1.07 to 21.30)) [54]. Therefore, caution should be taken when patients are already receiving treatment with a beta-blocker, rate-limited calcium-channel blocker, or other anti-arrhythmics. In patients with a resting heart rate less than 55 beats per minute (bpm) for ozanimod [65] or 50 bpm for etrasimod [66] or a history of myocardial infarction or heart failure, initiation should be performed with additional monitoring with hourly pulse and blood pressure measurements and a repeat ECG at 6 h (4 h for etrasimod) [65,66]. All cardiac abnormalities, including hypertensive crisis, developed within 48 h of treatment initiation in the clinical trials. In patients with risk factors, it is therefore also reasonable to repeat an ECG at 7 days.

### 9.5. Ophthalmological Assessment

Patients with a history of anterior uveitis, diabetes mellitus, or co-existing retinal disease should undergo an ophthalmological assessment prior to initiation of S1PRMs. If no risk factors are present, then an assessment should be performed within 3 months of initiation of therapy to screen for macular oedema.

### 9.6. Monitoring During Treatment and Managing Side-Effects

Liver function should be assessed at 1, 3, 6, 9, and 12 months and then annually according to the summary of product characteristics [65,66]. If liver transaminases above five times the upper limit of normal (ULN) are confirmed, or at least three times the ULN associated with an increase in serum bilirubin more than two times the ULN (Hy’s law [67]), treatment with S1PRM should be interrupted and only re-commenced once liver transaminase values have normalized (including if an alternative cause of the hepatic dysfunction is discovered) [65,66].

S1PRMs cause a reduction in peripheral lymphocyte count to approximately 45% of baseline values, due to reversible retention of lymphocytes in lymphoid tissues. Full blood count assessments are recommended periodically during treatment. If the lymphocyte count falls below 0.2 × 10^9^/L, the S1PRM should be held until the level reaches > 0.5 × 10^9^/L when re-initiation can be considered [65,66].

Should patients develop symptoms of macular oedema, such as blurred or distorted vision, urgent ophthalmological assessment is advised along with S1PRM cessation. Similarly, if hypertension, bradycardia, or symptoms of arrhythmia develop, the S1PRM should be held.

## 10. Future Directions

While clinical trials have demonstrated favourable short-term efficacy and safety of S1PRMs in IBD, knowledge gaps remain in long-term safety and real-world effectiveness, particularly in diverse and older populations. Issues such as infection, malignancies, cardiovascular complications, and hepatotoxicity over prolonged use are not yet fully characterized, and prospective studies will be essential to address this.

The results of phase 3 trials in CD are awaited, and additionally, the role of S1PRMs in treating extraintestinal manifestations and other IMIDs is currently unclear. There is promising early data for their utility in systemic lupus erythematosus [68], plaque psoriasis [69], Sjogren’s syndrome, and systemic sclerosis [70], and further data may yet demonstrate wider utility of this drug class.

Finally, there is growing interest in sequencing or combining S1PRMs with other advanced therapies, particularly in refractory or complex cases. Advanced combination therapy raises concerns about additive immunosuppression; however, the favourable safety profile of this drug class, particularly with regard to infection risk, makes it an attractive potential agent for this treatment strategy [71].

## 11. Conclusions

S1PRMs represent a novel class of agents that have proven efficacy in treating UC through modification of lymphocyte tracking. Early data suggests they may also be efficacious in CD, and ongoing phase 3 trials will look to confirm this. Their mechanism of action results in a minimal side-effect profile, and whilst there are some concerns with regard to bradyarrhythmia, hypertension, and macular oedema in certain individuals, they appear to be overall a low-risk class of drug. Future research should focus on longer-term outcomes, their efficacy in wider IMIDs, and potential use in advanced combination therapy alongside monoclonal antibodies or JAK inhibitors.

## Figures and Tables

**Table 1:** Downstream signalling molecules and function of S1P receptor subtypes. Adapted from Peyrin-Biroulet et al. [9]. IL: interleukin; S1P: sphingosine-1 phosphate; S1PR: S1P receptor.

Receptor	Expression	Biologic Outcomes	Clinical Relevance	Targeted Therapies
S1PR1	Broad, including B-cells, T-cells, dendritic cells, cardiac tissue, neurons	Lymphocyte migration, bradycardia	Autoimmune modulation, bradycardia	Etrasimod, ozanimod, fingolimod
S1PR2	Broad, including vascular smooth muscle, endothelium, cardiac tissue, lung fibroblasts	Vasoconstriction, inflammation, fibrosis, inhibition of B cell survival, proliferation	Renal injury, fibroblast contraction	Fingolimod
S1PR3	Broad, including vascular smooth muscle, endothelium, cardiac tissue, lung fibroblasts	Vasoconstriction, fibrosis, proliferation	Hypertension	Fingolimod
S1PR4	Restricted, only T cells, dendritic cells, breast cancer cells	Inhibition of effector cytokines, secretion of IL-10.	Autoimmune modulation	Etrasimod, fingolimod
S1PR5	Restricted, only natural killer cells, endothelial cells, oligodendrocytes	Natural killer cell migration, blood–brain barrier integrity, oligodendrocyte migration	Autoimmune modulation, myelination	Etrasimod, filgonimod, ozanimod

**Table 2:** Overview of the pharmacokinetics and pharmacodynamics of currently available Sphingosine-1-phosphate receptor modulators used for multiple sclerosis and ulcerative colitis (adapted from [29]). MS: multiple sclerosis; S1PR: Sphingosine-1-phopshate receptor; UC: ulcerative colitis.

	Fingolimod	Ozanimod	Etrasimod
Indication(s)	Relapsing remitting MS in adults and children aged ≥10 years	Relapsing remitting MS in adults Moderate-to-severe UC in patients who have had an inadequate response or loss of response or were intolerant to first-line therapies	Moderate-to-severe UC in patients aged ≥16 who had an inadequate response or loss of response or were intolerant to first-line therapies
Receptor selectivity	S1PR1, S1PR3, S1PR4, S1PR5	S1PR1, S1PR5	S1PR1, S1PR4, S1PR5
Oral bioavailablity	~93%	~60%	~90%
Time to peak (Tmax)	12–16 h	6–8 h	2–8 h
Half-life (_t1/2_)	6–9 days	Ozanimod: ~20 h CC112273: ~11 days	26–32 h
Time to steady-state	1–2 months	7 days	7 days
Time to lymphocyte reduction	4–6 h	6–12 h	1–3 h
Lymphocyte decrease from baseline	70%	34–68%	41–69%
Lymphocyte recovery after discontinuation	1–2 months	~3 months	1–2 weeks
Dose titration	Required	Required	Not required

**Table 3:** Summary of ozanimod trials in ulcerative colitis: TOUCHSTONE and TRUE NORTH. Efficacy outcomes defined as clinical remission (Mayo score ≤ 2, with no individual subscore > 1), clinical response (defined as a reduction from baseline in the Mayo Clinic score of ≥3 points and ≥30% and a decrease from baseline in the rectal bleeding subscore), and mucosal healing (endoscopy subscore of ≤1 point) [22,23]. AE: adverse event; ALC: Absolute lymphocyte count; mg: Milligram.

	TOUCHSTONE	TRUE NORTH
Phase	Phase 2	Phase 3
Sample size	197	645 (induction), 457 (maintenance)
Population	Moderate-to-severe ulcerative colitis	Moderate-to-severe ulcerative colitis
Trial design	1:1:1 randomization, 8-week induction, 32-week extension	2:1 randomization, 10-week induction, 42-week maintenance
Dosing	Ozanimod 0.5 mg, 1 mg, or placebo once daily	Ozanimod 0.92 mg or placebo once daily
Primary endpoint	Clinical remission at week 8	Clinical remission at week 10
Clinical remission	0.5 mg: 14% (9/65) 1 mg: 16% (11/67) Placebo: 6% (4/65)	-Week 10: 0.92 mg: 18.4% (79/429) vs. placebo: 6.0% (13/216) -Week 52: 0.92mg: 37% (85/230) vs. placebo: 18.5% (42/227)
Clinical response	0.5 mg: 54% (35/65) 1 mg: 57% (38/67) Placebo: 37% (24/65)	-Week 10: 0.92 mg: 47.8% (205/429) vs. placebo: 25.9% (56/216) -Week 52: 0.92 mg: 60% (138/230) vs. placebo: 18.5% (42/227)
Mucosal healing	0.5 mg: 28% (18/65) 1 mg: 34% (23/67) Placebo: 12% (8/65)	-Week 10: 0.92 mg: 12.6% (54/429) vs. placebo: 3.7% (8/216) -Week 52: 0.92 mg: 29.6% (68/230) vs. placebo: 14.1% (32/227)
ALC reduction	0.5 mg: −32%; 1 mg: −49%	−54%
Safety	The most common AEs were headache and anaemia. Singular case of transient sinus bradycardia and atrioventricular block.	Similar safety profile to TOUCHSTONE, higher incidence of bradycardia during induction

**Table 4:** Summary of etrasimod trials in ulcerative colitis: OASIS, ELEVATE-UC 52, ELEVATE-UC 12. Efficacy outcomes defined as clinical remission (Mayo score ≤ 2, with no individual subscore > 1), clinical response (defined as a reduction from baseline in the Mayo Clinic score of ≥3 points and ≥30% and a decrease from baseline in the rectal bleeding subscore), and mucosal healing (endoscopy subscore of ≤1 point). [26,33]. AE: adverse event; ALC: absolute lymphocyte count; AV: atrioventricular; ETR: etrasimod; PBO: placebo.

	OASIS	ELEVATE-UC 52	ELEVATE-UC 12
Phase	Phase 2	Phase 3	Phase 3
Sample size (*n*)	*n* = 156	*n* = 433 (maintenance)	*n* = 354
Population	Moderate-to-severe ulcerative colitis	Moderate-to-severe ulcerative colitis (responders)	Moderate-to-severe ulcerative colitis
Trial design	1:1:1 randomization, 12 weeks	2:1 randomization, 12 week-induction + 40 week maintenance + treat-through OLE	2:1 randomization, 12-week induction + treat-through OLE
Dosing	Etrasimod 1 mg, 2 mg, or placebo once daily	Etrasimod 2 mg or placebo once daily	Etrasimod 2 mg or placebo once daily
Primary endpoint	% change in modified Mayo score	Clinical remission at weeks 12 and 52	Clinical remission at week 12
Clinical remission	1 mg: 16%; 2 mg: 33% Placebo: 8.1%	Week 12: ETR 25% vs. PBO 15% Week 52: ETR 32% vs. PBO 7%	ETR: 26% Placebo: 15%
Clinical response	1 mg: 43.7%; 2 mg: 50.6%; Placebo: 32.5%	ETR (week 12): 62% PBO (week 12): 34%	ETR (week 12): 62% PBO (week 12): 41%
Mucosal healing	2 mg: 41.8% Placebo: 17.8%	ETR (week 12): 30% PBO (week 12): 14%	ETR (week 12): 33% PBO (week 12): 16%
ALC reduction	1 mg: −13.8% 2 mg: −39.9% Placebo: +17.3%	Sustained −60% to −65%	−64%
Safety	Mild AEs. Transient bradycardia; no serious infections or macular oedema reported	Transient bradycardia, 2 cases of AV block, 1 case of macular oedema	Transient bradycardia < 1%; 1 case of macular oedema

## Data Availability

Data sharing is not applicable to this article as no new data were created or analysed in this study.

## Abbreviations

Abbreviation	Meaning
AE (AEs)	Adverse event(s)
ALC	Absolute lymphocyte count
AV	Atrioventricular
bpm	Beats per minute
CD	Crohn’s disease
CDAI	Crohn’s Disease Activity Index
CCR7	C-C chemokine receptor type 7
CI	Confidence interval
ECG	Electrocardiogram
EMA	European Medicines Agency
ETR	Etrasimod
FDA	Food and Drug Administration
FBC	Full blood count
IBD	Inflammatory bowel disease
IL	Interleukin
IMID (IMIDs)	Immune-mediated inflammatory disease(s)
JAK	Janus kinase
kDa	Kilodalton
mg	Milligram
MS	Multiple sclerosis
NMA	Network meta-analysis
OD	Once daily
OLE	Open-label extension
PBO	Placebo
QTc	Corrected QT interval
RR	Relative risk
RWE	Real-world evidence
S1P	Sphingosine-1-phosphate
S1PR (S1PRs)	S1P receptor(s)
S1PRM (S1PRMs)	S1P receptor modulator(s)
SAE (SAEs)	Serious adverse event(s)
SES-CD	Simple Endoscopic Score for Crohn’s Disease
SphK1	Sphingosine kinase 1
SphK2	Sphingosine kinase 2
t1/2	Half-life
TEAE (TEAEs)	Treatment-emergent adverse event(s)
Tmax	Time to peak concentration
TNF (TNFs)	Tumour necrosis factor(s)
UC	Ulcerative colitis
ULN	Upper limit of normal
